# Formulation and production of a blood‐free and chemically defined virus production media for VERO cells

**DOI:** 10.1002/bit.27486

**Published:** 2020-08-01

**Authors:** Randall Alfano, Atherly Pennybaker, Peter Halfmann, Claire Y.‐H. Huang

**Affiliations:** ^1^ InVitria Junction City Kansas; ^2^ Division of Vector‐Borne Diseases Centers for Disease Control and Prevention Fort Collins Colorado; ^3^ Department of Pathobiological Sciences, Influenza Research Institute, School of Veterinary Medicine University of Wisconsin Madison Wisconsin

**Keywords:** plant‐derived hydrolysate, recombinant albumin, recombinant transferrin, serum‐free culture medium, VERO cells

## Abstract

Vaccines provide effective protection against many infectious diseases as well as therapeutics for select pathologies, such as cancer. Many viral vaccines require amplification of virus in cell cultures during manufacture. Traditionally, cell cultures, such as VERO, have been used for virus production in bovine serum‐containing culture media. However, due to concerns of potential adventitious agents present in fetal bovine serum (FBS), regulatory agencies suggest avoiding the use of bovine serum in vaccine production. Current serum‐free media suitable for VERO‐based virus production contains high concentrations of undefined plant hydrolysates. Although these media have been extensively used, the lack of chemical definition has the potential to adversely affect cell growth kinetics and subsequent virus production. As plant hydrolysates are made from plant raw materials, performance variations could be significant among different lots of production. We developed a chemically defined, serum‐free medium, OptiVERO, which was optimized specifically for VERO cells. VERO cell growth kinetics were demonstrated to be equivalent to EMEM‐10% FBS in this chemically defined medium while the plant hydrolysate‐containing medium demonstrated a slower doubling time in both two‐dimensional (2D) and 3D cultures. Virus production comparisons demonstrated that the chemically defined OptiVERO medium performed at least as good as the EMEM‐10%FBS and better than the plant hydrolysate‐containing media. We report the success in using recombinant proteins to replace undefined plant hydrolysates to formulate a chemically defined medium that can efficiently support VERO cell expansion and virus production.

## Graphical abstract

OptiVERO serum‐free, chemically defined medium supports growth kinetics and virus productivity equivalent or better than traditional media. Using recombinant proteins in VERO cell cultures reduces variability and increases efficiency in vaccine and therapeutics development. Comparisons to media containing undefined plant hydrolysates and fetal bovine serum, in both 2D and 3D cell cultures, are given.

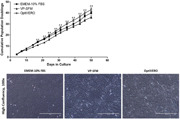

## 1 INTRODUCTION

Vaccines currently represent a versatile class of prophylactics in the defense of major infectious disease as well as novel class of therapeutics for serious conditions such as cancer (Ong, Tan, & Ho, 2017). Manufacturing of both live‐attenuated viral vaccine and inactivated vaccine made from inactivation of live virus requires production of a large amount of viruses from host cells. Recent years have witnessed a major shift in the paradigm of viral vaccine manufacturing from more primitive manufacturing platforms, such as embryonated chicken eggs and primary and diploid cell culture systems, to more robust large‐scale methods that utilize mammalian continuous cell line (CCL)‐based systems (Hegde, 2015). VERO (African monkey kidney epithelial) cells have been utilized for the manufacturing of human vaccines for nearly 40 years and were at one point, the only CCL approved by the World Health Organization (Barrett, Mundt, Kristner, & Howard, 2009). As a result, VERO is currently the most widely utilized and best characterized CCL for vaccine production (Hegde, 2015).

VERO cells have a multitude of inherent benefits to serve as a viral production cell line, including the inability to express interferon‐α that can hinder viral replication, low doubling time, and relative ease of use (Desmyter, Melnick, & Rawls, 1968). VERO cells grow readily in Dulbecco's modified Eagle's medium (DMEM) or Eagle's minimum essential medium (EMEM) containing 10% fetal bovine serum (FBS; EMEM‐10% FBS) and have been documented to produce a multitude of viruses for vaccine production at commercially viable levels (Barrett et al., 2009; Desmyter et al., 1968; Govorkova, Kodihali, Alymova, Fanget, & Webster, 1999). However, due to safety concerns, current guidance by regulatory bodies in addition to Good Manufacturing Practice compliant manufacturing of cell‐based products (advanced therapy medicinal products) states that alternatives to FBS should be utilized due to poor reproducibility and safety concerns associated with FBS use (EMA, 2011; van der Valk et al., 2017). As a result, several serum‐free media have been described for VERO‐based virus production and are currently available to the market (Merten, Kierulff, Castignolles, & Perrin, 1994; Price, 1997, 2002). These media have demonstrated excellent ability to support VERO cell growth and virus production (Price, 2002).

VERO cells can be readily adapted to serum‐free conditions but remain dependent on the major protein components in serum for successful propagation. Serum albumin provides multiple functions in vitro, including antioxidant, macromolecule delivery, and free radical scavenger functions (Lee & Wu, 2015; Taverna, Maria, Mira, & Guidet, 2013). Serum transferrin delivers iron to cells via catalytic cycling (Steere et al., 2012). Cytokines deliver the necessary mitogenic signaling to maintain the proliferation rate and promote survival of VERO cells. Some commercially available serum‐free media for VERO cells, such as VP‐SFM, contain very low levels of protein (typically 10 ng/ml or less) that includes recombinant human epidermal growth factor (EGF) as the only full‐length protein (Life Technologies, 1999). The EGF cytokine appears to be important for VERO expansion (Life Technologies, 1999). Furthermore, iron chelates can be utilized as an efficient substitute for serum transferrin, and albumin can be replaced with plant‐derived di‐ and tripeptides at a final concentration of 100–200 mg/L (Life Technologies, 1999). As a result, these plant hydrolysate‐containing media are void of any animal components and are thus considered to be animal free.

The ability of these media to support VERO cell proliferation is mostly dependent on the presence of these plant‐derived hydrolysates as withdrawal of this complex mixture results in cessation of cell proliferation (Mizrahi & Shahar, 1977). It is thought that protein hydrolysates act as a concentrated, balanced nutrient mixture that can at least in part replace serum (Jan, Jones, Emery, & al‐Rubeai, 1994). However, current understanding of these complex mixtures cannot unequivocally provide evidence that beneficial activity resides in a small fraction of the mixture or whether the beneficial activity observed is mostly derived from interactions of individual components in the mixture (Franek, Hohenwarter, & Katinger, 2000). Indeed, attempts to separate peptide fractions via chromatography identified fractions with significantly variable growth‐promoting activities in mammalian cell culture (Franek et al., 2000). Thus, media containing these undefined complex mixtures can complicate manufacturing efforts due to potential variable performance among different lots of the mixture made from poorly controlled raw material.

To circumvent the lack of chemical definition in virus production media, we have utilized recombinant human transferrin and recombinant human albumin to formulate a chemically defined serum‐free media that is optimized specifically for production of virus in VERO cells in both two‐dimensional (2D) and 3D culture formats. The efficacy of this chemically defined media, known as OptiVERO, is demonstrated in both the 2D and 3D expansion as well as the production of three different viruses in comparison to both serum and plant hydrolysate‐containing media. Overall, the data presented here provide proof of principle that poorly defined plant hydrolysates can be replaced with recombinant proteins to formulate a chemically defined, serum‐free virus production media suitable for clinical application.

## 2 MATERIALS AND METHODS

### 2.1 Cells

VERO cells used for cell propagation experiments were originally obtained from American Type Culture Collection (ATCC; CCL‐81; Manassas, VA). VERO cells were routinely cultured in flasks (Corning, Corning, NY) at 37°C/5% CO_2_ in humidified incubators and passaged every 3–5 days. Dengue and Zika virus (DENV and ZIKV) production evaluations were performed using VERO cells originally obtained from Sigma‐Aldrich (St Louis, MO) and maintained at the Centers for Disease Control and Prevention (CDC). A VERO‐VP30 cell line that stably expresses the VP30 gene of *Zaire ebolavirus* was established as previously described (Halfmann et al., 2008) for amplification of a biologically contained EbolaΔVP30 virus, and a working cell bank of this cell line was then established by Waisman Biomanufacturing (Madison, WI).

### 2.2 Reagents

Cellastim S, recombinant human serum albumin derived from a nonmammalian host and Optiferrin, recombinant human transferrin derived from a nonmammalian host (InVitria, Junction City, KS) were reconstituted according to the manufacturer's instructions. Sterile‐filtered liquid stocks were maintained at 4°C for 1 month. EMEM (MediaTech, Manassas, VA) supplemented with FBS (ATCC, Manassas, VA) was used as the traditional FBS‐containing medium control in cell expansion and virus culture experiments. VP‐SFM (Thermo Fisher Scientific, Waltham, MA) was supplemented with 4 mM Glutamine (Thermo Fisher Scientific) and is referred as VP‐SFM throughout the text.

### 2.3 Formula development

Media formulations were designed using a design of experiments (DOE)‐based approach. For initial reagent screening, 23‐factor Placket Burman matrices were utilized. Subsequent refinement of the medium formulation was performed using central composite designs using factors identified as being statistically significant drivers of VERO cell growth in screens. Briefly, variable reagents were formulated as a 10× concentrate using a predetermined DOE design. Nonvariable components were formulated into a base medium, DMEM/F12 (Thermo Fisher Scientific). The base medium (0.24 ml/well) was added to 48‐well plates (VWR, Radnor, PA) followed by the addition of 0.03 ml/well of the 10× variables in triplicate. EMEM‐10% FBS and VP‐SFM were utilized as interplate controls. VERO cells were extensively washed with Dulbecco's phosphate‐buffered saline (DPBS; GE, Fairfield, CT) and detached using 1× TrypLE (Thermo Fisher Scientific) with 1 mM ethylenediaminetetraacetic acid (EDTA; Sigma‐Aldrich). After the cells were detached, 0.25% Soybean Trypsin Inhibitor (Thermo Fisher Scientific) in DPBS was used to neutralize the TrypLE and cells were collected by centrifugation. Cells were resuspended in basal DMEM/F12 and seeded (0.03 ml/well) into the 48‐well plates containing formulated medium at an initial density of 5,000 cells/cm^2^. Cells were incubated in standard culture conditions as described above for ∼96 hr and subsequently harvested by trypsinization and counted on a pregated bench top flow cytometer (Guava; EMD Millipore, Burlington, MA). For Placket Burman‐based designs, data were analyzed via linear regression and significance of means was determined using a *t* test. For central composite designs, polynomials to model the second‐order response surface were generated and subsequently verified by the same experimental method described above.

### 2.4 Two‐dimensional multiple cell‐passage expansion

Early passage VERO cells maintained in EMEM‐10% FBS were extensively washed with DPBS to remove all traces of serum and subsequently removed from the growth surface using 0.05 ml/cm^2^ TrypLE containing 1 mM EDTA. Equal volume of 0.25% soybean trypsin was added, and cells were collected with the addition of 0.2 ml/cm^2^ DPBS. Cells were centrifuged at 200*g* for 5 min and resuspended in a minimal volume of prewarmed OptiVERO SFM (our chemically defined medium). Cells were seeded in T‐75 flasks containing prewarmed EMEM‐10% FBS, VP‐SFM containing 4 mM l‐glutamine, or OptiVERO SFM at an initial cell density of 10,000 cells/cm^2^. VERO cells were subcultured every 3–5 days until a total of 14 serial passages was attained. At each passage, population doublings were determined in each media formulation. Cumulative population doublings were determined and presented.

### 2.5 Microcarrier‐based (3D) cell expansion

VERO cells were adapted to VP‐SFM with 4 mM l‐glutamine or the chemically defined media for at least three passages in T‐flasks before 3D expansion on microcarriers. Briefly, cells were expanded in multiple T‐150 flasks to achieve sufficient cells for microcarrier seeding. The day before seeding, untreated plastic microcarriers (Corning) in cell culture grade water (1,000 mg/ml; Sigma‐Aldrich) and glass spinner flasks (Corning) were sterilized and allowed to cool overnight. On the day of cell inoculation, sterilized microcarriers were washed three times in EMEM to remove all traces of water. Cold VP‐SFM, EMEM‐10% FBS, or our developed OptiVERO SFM was used on a final wash before seeding the microcarriers in the spinner flask containing respective medium. Microcarriers (4,200 mg) were added per spinner flask to generate a 10 cm^2^/ml microcarrier suspension for a total of 1,500 cm^2^ per spinner flask. The spinner flasks were allowed to equilibrate at 37°C for a minimum of 3 hr before cell inoculation. VERO cells were trypsinized and collected from the T‐150 flasks using standard methods as described above. Cells were resuspended in 5 ml of each testing medium, passed through a 0.7 µm nylon cell strainer (Corning), and counted. Cells were adjusted to 1 × 10^6^ viable cells/ml in each prewarmed growth medium and 15 ml of each cell suspension was added to the equilibrated spinner. Spinners were returned to the stir plate set at 40 rpm in a 37°C 5% CO_2_ incubator for 48 hr. Starting at Day 2, spinners were monitored every day for cell growth, culture glucose, and pH. Cultures with glucose levels below 2 g/L were adjusted back up to 2 g/L using 45% glucose (Sigma‐Aldrich) and pH was restored to neutral with 7.5% sodium bicarbonate (Thermo Fisher Scientific). Cell growth was determined by treating the microcarriers in a lysis buffer solution containing 10 mM Sodium Citrate (Sigma‐Aldrich) and 1% Triton X‐100 (Sigma‐Aldrich). Nuclei were harvested, stained (Viacount nuclear stain EMD Millipore), and counted on a pregated flow cytometer (Guava; EMD Millipore). Spinner runs were terminated when glucose consumption ceased (about 9 days).

### 2.6 Virus production

Dengue serotype 2 virus, strain 16681 (DENV‐2 16681), and Zika virus, strain PRVABC59 (ZIKV PRVABC 59) were available at Division of Vector‐Borne Diseases (DVBD), CDC (Fort Collins, CO). A biologically contained *Zaire ebolavirus*, EbolaΔVP30 virus, possessing the green fluorescent protein reporter gene, instead of the VP30 gene in the viral genome, was generated as previously described (Halfmann et al., 2008). EbolaΔVP30 virus is approved for use under biosafety level‐2 containment solely at the University of Wisconsin‐Madison by the NIH and is exempt from the CDC Select Agent registration.

For DENV‐2 16681 and ZIKV PRVAB59 production, VERO cells maintained in DMEM‐10% FBS at CDC laboratory were adapted to either VP‐SFM, EMEM‐10% FBS, or OptiVERO SFM via three serial passages in T‐150 flasks before virus infection in T‐flasks (2D culture) or 150 ml microcarrier/spinner (3D) cultures. For flask‐based infections, cells were allowed to grow up to subconfluence (3 days) before infection while microcarrier/spinner flasks were cultured for 3–4 days. On the day of infection, 100% (T‐flasks) or 75% (spinner flasks) of the growth medium was removed and virus was introduced at 0.001 PFU/cell in basal EMEM. Cells were incubated at 37°C for 90 min in minimal volume for virus adsorption, followed by the addition of fresh prewarmed medium (VP‐SFM, EMEM‐10%, or OptiVERO SFM). Virus was allowed to propagate for 8–10 days with sample aliquots being removed for plaque titration every day or every other day depending on the virus and stored at −80°C until analysis. Culture glucose was maintained at 2 g/L and neutral pH via the addition of a 45% glucose solution (Sigma‐Aldrich) and 7.5% sodium bicarbonate (Thermo Fisher Scientific). Virus production of EbolaΔVP30 virus in 2D culture was performed in a similar manner with the following changes. VERO‐VP30 cells of the working cell bank (Passage 5) were seeded in T‐75 flasks. On the following day, old medium was replaced with fresh prewarmed medium containing virus at an infection of 0.001 PFU/cell. Virus was allowed to propagate and sample aliquots (1 ml) were harvested on Days 3, 6, and 9 and frozen at −80°C for virus plaque titrations.

### 2.7 Virus titration

DENV‐2 and ZIKV titration to measure plaque forming unit was performed in 6‐well plates of just confluent VERO cells as described previously (Huang et al., 2000). Briefly, virus samples were thawed on ice and subjected to 10‐fold serial dilutions in basal DMEM media. Growth media was removed from the confluent VERO monolayers and 100 µl of diluted virus was added per well. Inoculated cell plates were incubated at 37°C for 2 hr with gentle rocking of the plates every 15 min. Cells were overlayed with 4 ml overlay medium containing 0.8% SeaKem LE agarose. Plates were stored upside down at 37°C CO_2_ incubator for 4–7 days (DENV‐2 16681) or 4–5 days (ZIKV PRVABC 59) before adding a second overlay (2 ml/well) containing 4.8% of 1:300 neutral red solution (MP Biomedicals, Santa Ana, CA). Plates were incubated for an additional 24–48 hr and virus plaques were counted for three consecutive days. For EbolaΔVP30 viruses, VERO‐VP30 cells were plated in 12‐well plates in MEM + 10% FBS and allowed to grow to confluence. On the day of infection, virus samples were thawed on ice and subjected to 10‐fold dilutions in basal EMEM. Growth medium was removed from the confluent VERO‐VP30 monolayer and 100 µl of diluted virus was added per well. Cells were incubated at 37°C for 60 min with gentle rocking of the plates every 15 min. Cells were washed twice with PBS, 1.5 ml of 1.25% methylcellulose media overlay [18] was added to each well, and cells were incubated at 37°C for 1 week. After fixation with 10% neutral formalin, plaques were visualized as previously described (Halfmann et al., 2008).

### 2.8 Statistics

One‐way analysis of variance with 95% confidence interval was utilized to determine the significance of differences in means between different media.

## 3 RESULTS

### 3.1 Formulation of the animal component‐free chemically defined virus production media

**Figure 1:**
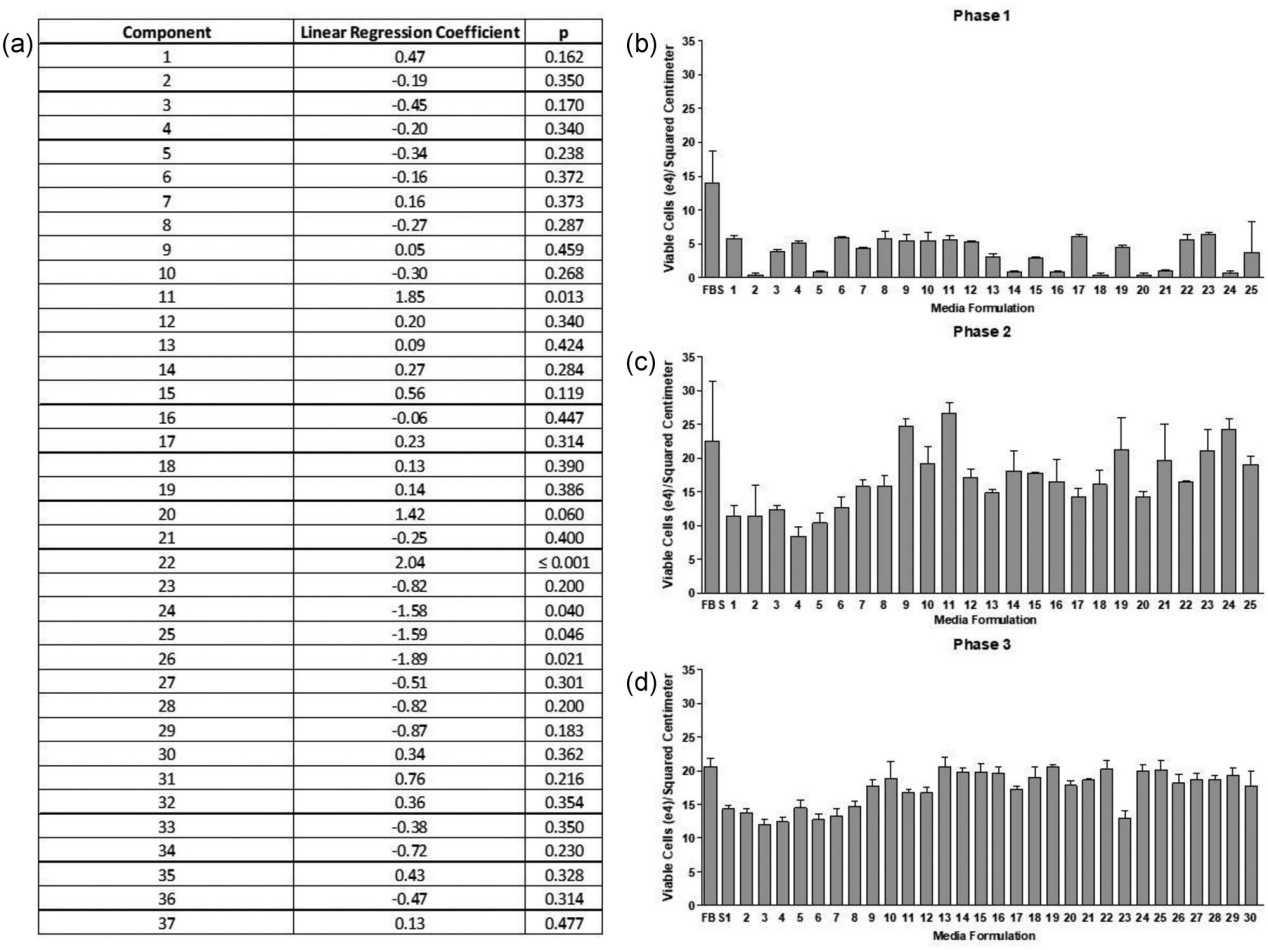
Formulation of the VERO‐optimized ACF media. Medium components (37 total) were first screened in basal DMEM/F12 to identify approximate optimal concentration ranges. Plackett–Burman matrix designs were then employed to determine optimal reagent mixtures in these identified concentration ranges using VERO cell proliferation in 48‐well plates as the readout. (a) Linear regression analysis was performed on the 37 different components in Phase 1 using two separate Plackett–Burman experiments. Components 11 and 22 were identified as significant drivers of VERO cell proliferation with a linear regression slope of 1.85 and 2.04, respectively (*p* ≤ .01 and .001). Component 20 induced a strong yet insignificant induction of proliferation in VERO cells. Components 24–26 induced a significant inhibition of VERO cell proliferation as evidenced by highly negative slopes in linear regression analysis. (b) Representative data from Phase 1 Plackett–Burman matrix screening experiments. (c) For Phase 2, reagents from the 37 originally screened components were added and removed, and remaining component concentrations identified as significant in Phase 1 were optimized via steepest ascent/descent approaches. (d) Phase 3 employed central composite designs that optimized factor interactions in the media. DMEM, Dulbecco's modified Eagle's medium; FBS, fetal bovine serum

To formulate the chemically defined media, 37 different components (Figure 1a), including the recombinant albumin (Cellastim S) and recombinant transferrin (Optiferrin), were first titrated against VERO cells in basal DMEM/F12 to identify approximate concentration ranges that would be optimal for VERO cells. Plackett–Burman matrix designs were then utilized to screen novel formulations within the optimal concentration range of each component and linear regression analysis was performed. In total, two separate screens were performed in succession that were aimed at removing biologically inert or detrimental compounds using VERO cell growth as the readout (Figure 1a). A representative screen is shown in Figure 1b. Subsequent steepest ascent (Figure 1c) and central composite designs (Figure 1d) were employed to modify the concentration of select reagents as well as to identify and further leverage component interactions in the media formulation. To complete the media formulation work, a total of six candidates were formulated at small scale based on the quadratic model produced by the central composite design and confirmed via T‐flask‐based expansion versus EMEM‐10% FBS and VP‐SFM. From these early studies, a single formulation, referred to as OptiVero, was selected based on formulation complexity and performance for further cell growth kinetics and virus production experiments.

### 3.2 VERO cell expansion and morphology in OptiVERO

**Figure 2:**
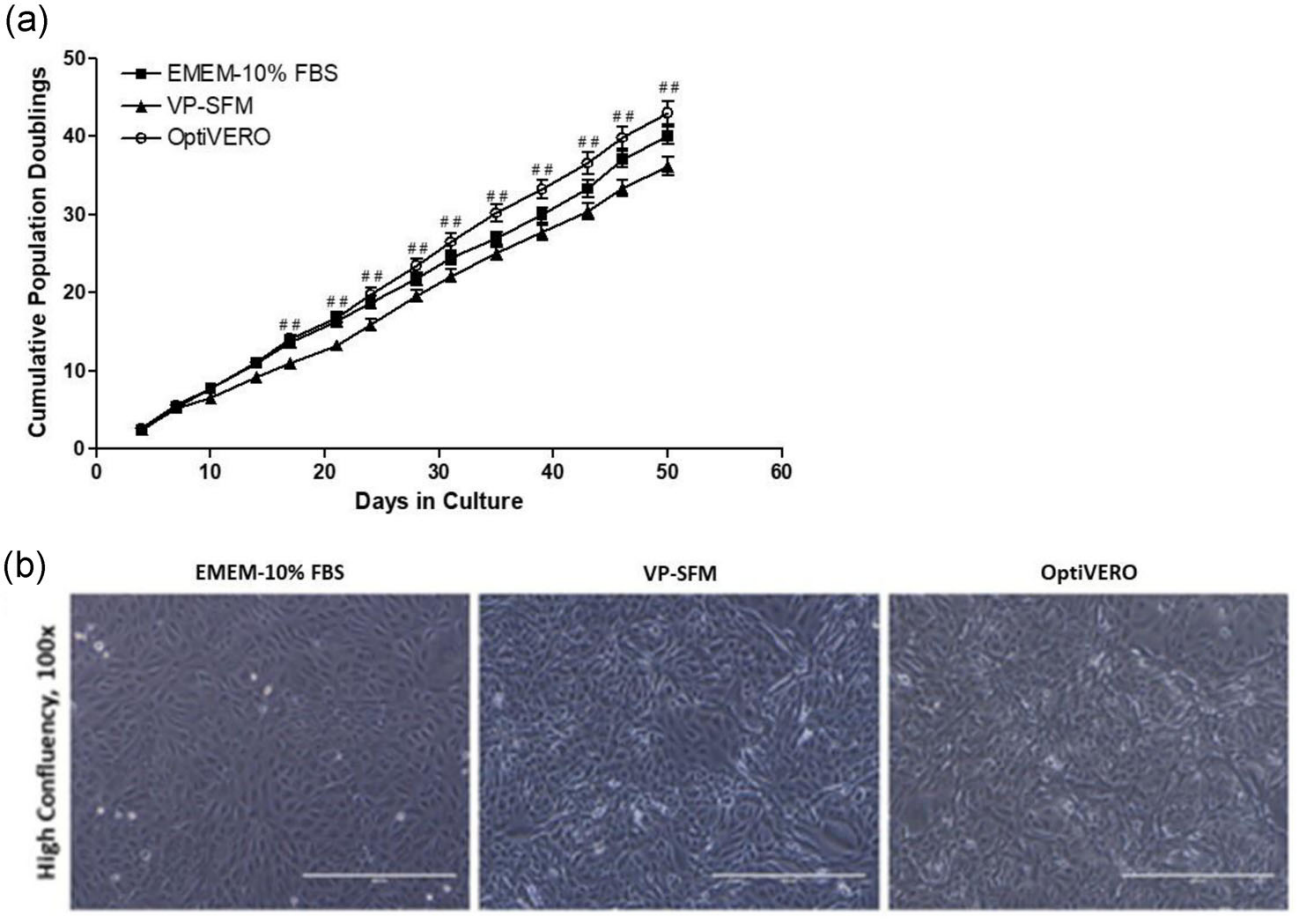
Growth kinetics and morphology of VERO cells in cell culture flasks. (a) VERO cells cultured in T‐75 flask with EMEM‐10% FBS (▪), VP‐SFM/4 mM Glutamine (Δ), or OptiVERO (○). Cells were subjected to a 14‐passage test in each medium and cumulative population doublings were determined over the 50‐day experimental period. ^##^Significant titer differences between OptiVERO and VP‐SFM as determined by one‐way ANOVA (b) morphology of cells cultured in OptiVERO appeared to be comparable to cells cultured in either 10% FBS or VP‐SFM. ANOVA, analysis of variance; EMEM, Eagle's minimum essential medium; FBS, fetal bovine serum [Color figure can be viewed at wileyonlinelibrary.com]

The serum‐free OptiVERO medium was subjected to a 14‐passage test in T‐75 flasks, comparing the population doublings at each passage to that of VP‐SFM and EMEM‐10% FBS. Cells cultured in EMEM‐10%FBS demonstrated a robust expansion capability, doubling 40.07 ± 4.16 times in the 50 days that the experiment was conducted. Similarly, OptiVERO exhibited 42.99 ± 5.68 doublings while VP‐SFM was significantly less robust, doubling only 36.15 ± 4.44 times, *p* ≤ .01 (Figure 2a). Cells cultured in the chemically defined serum‐free OptiVERO medium appeared to morphologically normal as compared to cells cultured in either EMEM‐10% FBS or VP‐SFM (Figure 2b).

**Figure 3:**
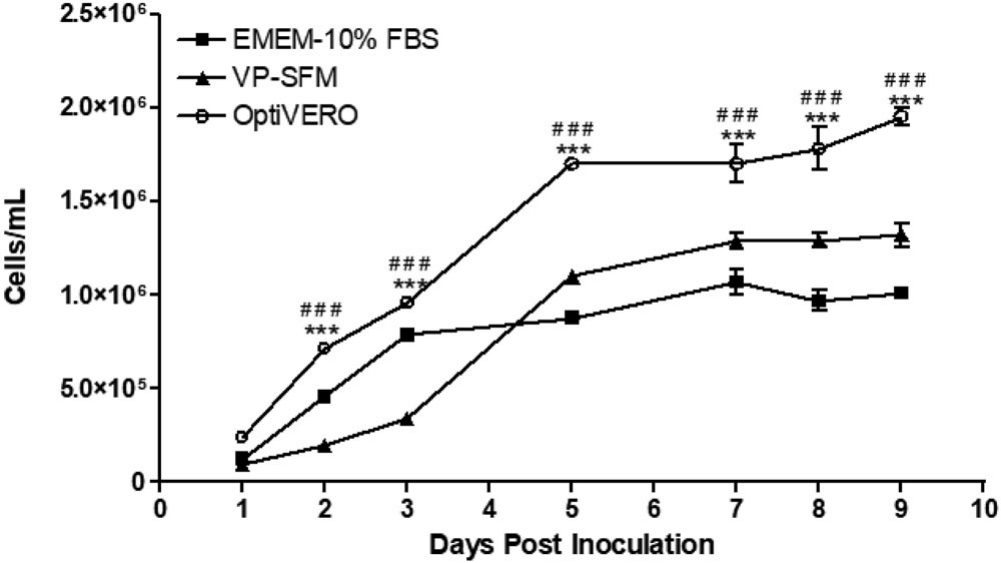
Growth kinetics of VERO cells expanded in OptiVERO in 3D culture. VERO cells were adapted to each tested medium for at least three passages in T‐flasks before microcarrier runs. Cells were seeded at an initial density of 10,000 cells/cm^2^ at Day 0 in 150 ml spinners. At Day 2 postseeding, spinners were monitored every day for cell growth, culture glucose, and pH. When necessary, cultures were fed with 45% glucose and 7.5% sodium bicarbonate to maintain the culture glucose at 2 g/L and pH neutral. VERO cells cultured in OptiVERO readily attached to the microcarriers and demonstrated a significantly higher cell expansion than either EMEM‐10% FBS (***) or VP‐SFM (^###^) as determined by one‐way ANOVA. Data are a representative run of at least four independent experiments. 3D, three‐dimensional; ANOVA, analysis of variance; EMEM, Eagle's minimum essential medium; FBS, fetal bovine serum

In the 3D cultures, VERO cells cultured in OptiVERO or EMEM‐10% FBS readily attached to plastic microcarriers and expanded rapidly within the first 3 days of spinner culture as evidenced by elevated glucose consumption and lactate production when seeded at low densities. VP‐SFM with 4 mM glutamine, although supporting VERO cell growth on plastic microcarriers, demonstrated a slower cell expansion rate in the first 3 days. In fact, the cell numbers in OptiVERO spinner were all significantly higher from 2 to 9 days after cell seeding (Figure 3).

### 3.3 Virus production in VERO cells cultured with OptiVERO

**Figure 4:**
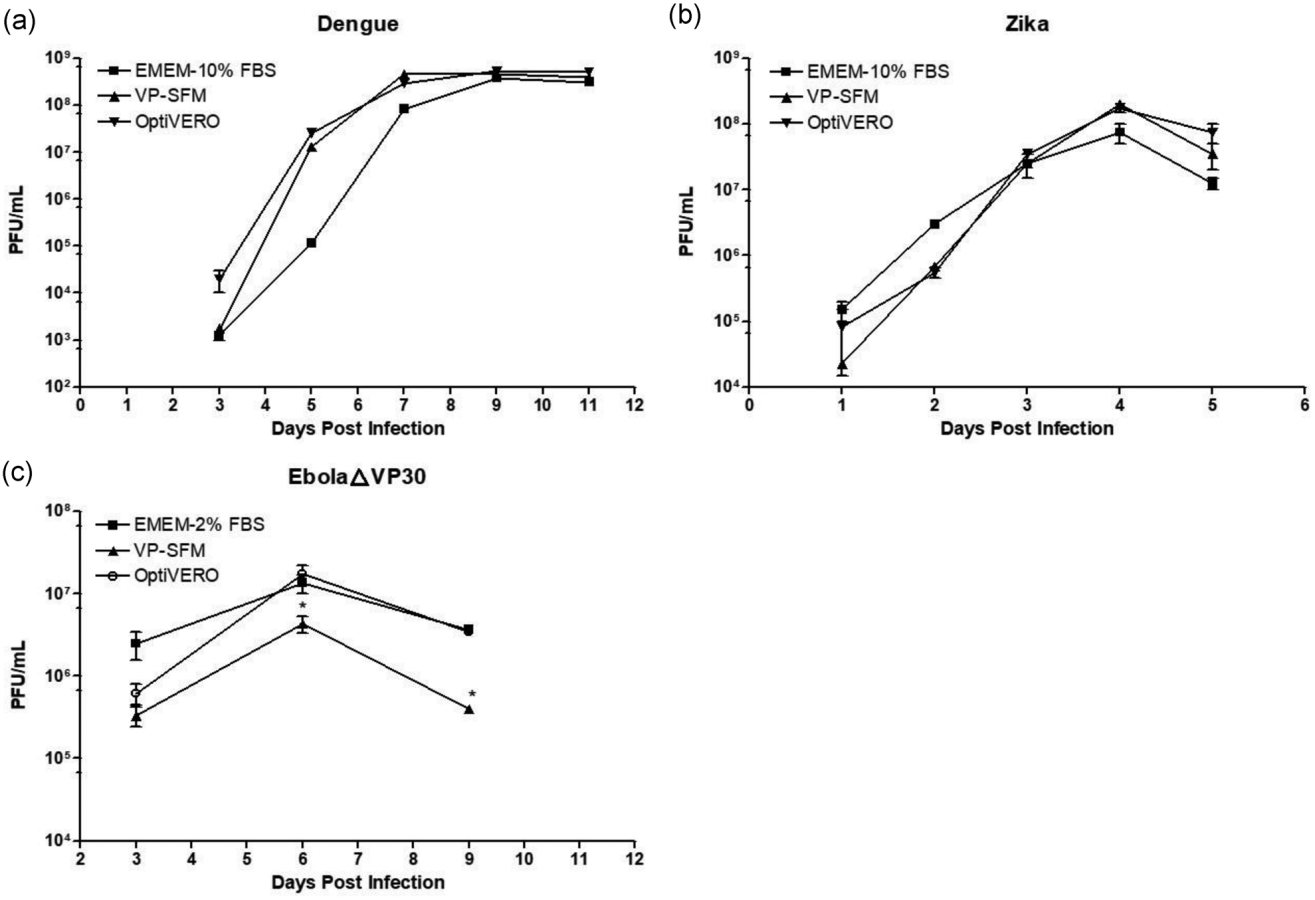
Virus production of VERO cells expanded in OptiVERO. (a) DENV‐2 16681 and (b) ZIKV PRVABC59 infected VERO cell cultures in 150 ml spinner with microcarriers (3D culture). The three media exhibited equivalent expansion of DENV‐2 16681 (a) and ZIKV (b) viruses. (c) EbolaΔVP30‐infected VERO cells in 2D T‐flask. VP‐SFM demonstrated a significant 1log reduction in virus titer compared to either OptiVERO or EMEM‐5% FBS, and the difference was consistent in three different experiments. *p ≤ .05 by one‐way ANOVA. Data of each virus run are representative of three independent production runs. 3D, three‐dimensional; ANOVA, analysis of variance; DENV, dengue virus; EMEM, Eagle's minimum essential medium; FBS, fetal bovine serum; ZIKV, zika virus

Two different flaviviruses, DENV‐2 16681 and ZIKV, and EbolaΔVP30 were used as proof of concept to determine the efficiency of OptiVERO in supporting virus production from VERO cells. VERO cells cultured in OptiVERO were highly competent in the production of both DENV‐2 and ZIKV by both 2D and 3D culture formats. The DENV‐2 was determined to be *3.7e8*, 4.6e8, and 5.3e8 PFU/ml for EMEM‐10% FBS, VP‐SFM, and OptiVERO, respectively, on Day 9 postviral infection (pi) in microcarrier/spinner cultures (13 days after microcarrier/spinner cell seeding; Figure 4a). Similarly, ZIKV titers were found to be 7.5e7, 2.0e8, and 1.75e8 PFU/ml for the three media on Day 4 pi of the 150 ml spinner 3D culture (Figure 4b). No significant difference in overall virus production yield among the three different culture media was demonstrated for either DENV‐2 or ZIKV. Trends were similar, albeit with less overall virus yield, in T‐flask (2D) culture (data not shown). For EbolaΔVP30, VERO‐VP30 cell line exhibited robust amplification of the virus plateauing on Day 6 pi with 1.35e7 PFU/mL and 1.74e7 PFU/mL, in EMEM‐2% FBS and OptiVERO, respectively. VP‐SFM demonstrated a significant 1 log reduction in virus titer (4.29e6 PFU/mL) and was consistent in three different experiments.

## 4 DISCUSSION

VERO cells are currently the best characterized CCL and have been extensively utilized for manufacturing of human vaccines for nearly 40 years (Barrett et al., 2009). Though VERO cells have a multitude of inherent benefits, they still require the presence of serum proteins to efficiently propagate. FBS, although extremely versatile, has significant issues with both a lack of reproducibility due to variation of composition as well as reliability of supply (van der Valk et al., 2017). It has been shown that different geographical and manufacturing lots of FBS can exhibit as large as 10‐fold difference in known components, in addition to the absence or presence of undefined substances (Baker, 2016a, 2016b). Growing demand for FBS across different areas of the biotech industry has put a severe strain on supply and resulted in a drastic price increase over the past decade. These conditions in combination with slack regulations are fertile grounds to encourage unethical practices and even blatant misconduct. Indeed, adulteration and fraud have been well noted in the serum industry. In 1994, 30,000 L of FBS were documented to be sourced from New Zealand; however, the country itself only reported a 15,000 L annual production (van der Valk et al., 2017). A second major adulteration case in 2013 was documented with the dilution of FBS with bovine albumin, water, and other growth‐promoting substances by a major global supplier (Gstraunthaler, Lindl, & van der Valk, 2014). Investigations by the Food and Drug Administration concluded that ∼280,000 L of FBS were affected between 2008 and 2013 (Gstraunthaler et al., 2014). In addition, animal products could potentially contain undesirable or dangerous adventitious agents, which is a safety concern of using FBS in vaccine manufacturing.

Although the issues with FBS usage, especially in the context of manufacturing of the clinical product, are well known, the component proteins that FBS contains are critical drivers of VERO cell growth, survival, and ultimately virus production. Serum albumin, one of the most abundant proteins in plasma of vertebrates, has multiple roles in several different biological processes (Lee & Wu, 2015). Likewise, serum transferrin can reversibly bind Fe^3+^ with nanomolar affinity and is one of the major vehicles for iron delivery to cells (Steere et al., 2012; Zhang et al., 2012). Thus, to have successful propagation of these cells in vitro, it is critical to incorporate proteins or compounds that can effectively substitute these bovine serum proteins. Human cell propagation has utilized human serum‐purified components to substitute for these bovine proteins and has fueled the xeno‐free marketing revolution in serum‐free media (Oikonomopoulous et al., 2015). However, use of protein derived from human serum does not mitigate the risk inherent in the variability of the serum source, limited supply availability, and risk of adventitious agent contamination. In addition, VERO cells are not of human origin and thus utilization of human serum‐derived proteins does not technically fall within the realm of “Xeno” free.

Plant‐derived di‐ and tripeptides offer another cost‐effective and technically feasible solution to replace these bovine or human serum‐derived proteins. Serum was shown to be effectively reduced to ∼20% of the original incorporation level by use of vegetable proteins in BHK cells, though full serum withdrawal demonstrated a significant reduction in cell expansion capacity (Mizrahi & Shahar, 1977). Further, peptone (0.5–2% wt/vol) was noted to effectively propagate multiple cell types in multiple media formulations (Taylor & Parshad, 1977). However, these mixtures are extremely complex in their composition and the mechanistic contribution to produce the observed positive effects in mammalian cell culture media is poorly understood (Franek et al., 2000). Fractionation attempts via low‐pressure liquid chromatography of both soy protein and wheat gluten demonstrated a significant spread of growth promotion and cell productivity using a murine hybridoma model (Taylor & Parshad, 1977). Furthermore, the inclusion of these complex ingredients in cell culture media can add unpredictability, such as the presence of unknown compounds that can potentially interfere with certain virus productivity.

Here, the protein components of FBS have been replaced with recombinant human serum albumin and recombinant human serum transferrin. These recombinant proteins were expressed in a nonmammalian expression platform that exhibits robust expression and exceptional scalability, and have been proven to be equivalent to their native counterparts both biochemically and functionally (Zhang et al., 2012). Recombinant transferrin (Optiferrin) was shown to possess the correct sequence to serum‐derived transferrin as determined by amino N‐terminal sequencing and the correct molecular weight as determined by Matrix‐Assisted Laser Desorption Ionization analysis (Steere et al., 2012; Zhang et al., 2012). Furthermore, the recombinant transferrin was able to both sequester and release iron as determined by in vitro iron‐binding and exhibited equivalent growth‐promoting activities of a murine hybridoma cell line as native human transferrin (Steere et al., 2012). Likewise, recombinant albumin (Cellastim S) has demonstrated equivalent biochemical and functional characteristics to the native version (Pennybaker & Alfano, 2018). Reverse‐phase analytical chromatography (RP‐UPLC) and liquid chromatography‐mass spectrometry (LC‐MS) demonstrated that the recombinant albumin contained the unmodified albumin species (with unmodified Cys‐34) and modified albumin species (with cysteinylated Cys‐34). These species were also present in human native albumin samples from commercial vendors (Pennybaker & Alfano, 2018). However, intact LC‐MS analysis and RP‐UPLC analysis demonstrated substantial variability in the amount of unmodified and modified species in the serum‐derived albumins. The ratio between unmodified and modified albumin species was shown to be highly conserved from lot to lot of recombinant albumin (Pennybaker & Alfano, 2018). In addition, the recombinant albumin was able to effectively expand primary human T cells that were comparable to native commercial preparations (Pennybaker & Alfano, 2018). The use of these recombinant proteins in cell culture virtually eliminates the risk of animal origin adventitious agent contamination, providing a significant advancement for the future development of the industry.

In conclusion, two highly scalable recombinant proteins, Cellastim S and Optiferrin, were utilized to replace the bovine components provided by serum to develop serum‐free medium candidates for VERO cell culture. Formulations were subsequently designed around these two components in addition to recombinant human EGF, to provide adequate nutritional and mitogenic support specifically for VERO cell expansion. Further optimization work of both the protein and nonprotein components within the formulation produced a chemically defined blood component‐free media, OptiVERO. This media supports rapid VERO proliferation in both 2D and 3D culture formats with a significantly lower doubling time than VP‐SFM. Furthermore, this media supported robust viral replication in VERO cell cultures for multiple viruses. The chemically defined OptiVERO can enhance the safety and consistency of virus production during manufacturing leading to improved virus‐based vaccines and therapeutics.
